# What implementation strategies are relational? Using Relational Theory to explore the ERIC implementation strategies

**DOI:** 10.3389/frhs.2022.913585

**Published:** 2022-10-17

**Authors:** Leah Bartley, Allison Metz, W. Oscar Fleming

**Affiliations:** ^1^Kaye Implementation and Evaluation, Tacoma, WA, United States; ^2^School of Social Work, University of North Carolina at Chapel Hill, Chapel Hill, NC, United States; ^3^School of Public Health, University of North Carolina at Chapel Hill, Chapel Hill, NC, United States

**Keywords:** implementation strategies, relational strategies, transactional strategies, Relational Theory, implementation practice

## Abstract

The identification and use of implementation strategies in implementation research and practice have strengthened our understanding of the implementation process as well as the causal pathways between mechanisms, strategies, and implementation outcomes. Although these contributions have advanced the application of strategies, there is still a need to learn more about how strategies might integrate relational exchanges and interactions. The inclusion of critical perspectives has been limited in implementation science, and theories such as Relational Theory can expand our understanding of the relational nature of implementation and enhance rigor through alternative theoretical applications. This study applied Relational Theory through a qualitative directed content analysis of the 73 Expert Recommendations for Implementation Change (ERIC) implementation strategies and examine relational components in strategy descriptions. Three reviewers used the structured approach to review and categorize the implementation strategies based on the Relational and Transactional Strategy Continuum measure, which operationalizes types of interactions, exchanges and alliances. Relational alliance strategies are those in which there is mutual growth and accountability, frequent interaction, shared power, and potential vulnerability. Operational alliances include forms of working exchanges between parties with balanced transactional and relational features. Operational alliances can be somewhat interactive in nature, with minor exchanges and limited accountability. Transactional alliance strategies are mostly uni-directional, influenced by power differentials, and do not require mutual growth, commitment, or exchange; thus, the power of growth is inherently one-sided. Results from the review suggest more implementation strategies with relational alliance features (highly relational, *n* = 17, semi-relational, *n* = 19) compared to transactional (highly transactional, *n* = 9, semi-transactional, *n* = 10) and 18 strategies coded as operational alliances. The qualitative review revealed opportunities to further expand how relational exchanges are considered within the implementation strategies descriptions, as well as the role of actors and power dynamics within strategy exchanges. The Relational and Transactional Strategy Continuum measure can help practitioners and researchers consider the sequencing, pairing, and impact on outcomes of different types and combinations of strategies in implementation practice and research. Additionally, the measure can support reflection on strategies that promote positive alliances, frequent connections, bi-directional communication, and power sharing.

## Introduction

The identification of the 73 Expert Recommendations for Implementation Change (ERIC) implementation strategies (1) in implementation science has contributed to the development of multiple taxonomies. To date, there have also been over 132 systematic reviews of discrete strategies to further identify and test strategies in implementation research (2). Strategies have also been further developed into causal pathway models, specifying the mechanisms and linkages that impact implementation success, though the limited application of theory to understand strategies' impact has contributed to suboptimal outcomes (3). There is an opportunity in implementation science to further expand the consideration and application of implementation strategies without limiting rigor (4). Implementation strategies continue to be trialed in service as usual (5, 6), and there is an opportunity to empirically understand how often implementation strategies occur in usual care. The inclusion of critical perspectives, such as feminist-based theories like Relational Theory, is also limited in implementation science. Critical perspectives offer the opportunity to expand the field's use of more flexible, reflexive, and critical approaches to understand the nuances of implementation in a variety of settings (7). Relational Theory posits that improvement and development in organizations and system change efforts are optimized in the context of connection and relational interactions. These interactions are characterized by mutual empathy and mutual empowerment (8). A relational perspective assumes that both conscious and unconscious phenomena are influenced primarily by relationships and acknowledges the power imbalances that may be present in mainstream culture, which places limited value on interdependence and vulnerability (9). While there is a growing focus on the role of relationships in implementation (10, 11), there is an opportunity to further understand the relational nature of implementation strategies used in practice and research (13, 14). Effective implementation support in research and practice, which includes the selection and tailoring of implementation strategies, is often dynamic and highly relational in nature because it involves multiple layers of context and differing norms and values among stakeholders.

At a clinical level, relational perspectives attend to how an individual has been shaped by negative and positive relations and interactions (9). Research has demonstrated that the strength and quality of relationships between patient and clinician (15), supervisor and worker (16), child and youth in foster care and worker (17) significantly impact the outcomes of the recipient of support. In an implementation study to understand enhanced patient decision support interventions in clinical practice through Relational Coordination theory, a sister theory to Relational Theory, high-performing clinics exhibited frequent, timely, accurate, and positive working relationships, which improved implementation of the patient decision support intervention (18). Another study of professionals who provide implementation support (11) reiterated relationships as critical for effective implementation, as respondents emphasized trust and mutuality as foundational for implementation efforts.

There is room to increase implementation science and practice by further defining and verifying relational techniques. Relational strategies include implementation strategies specifically designed to strengthen relationships and build trust among implementation stakeholders, as well as any implementation strategy that relies on mutual interaction to be successful in achieving implementation outcomes. As the field of implementation science seeks to identify mechanisms of change related to specific strategies, studying relational aspects of the implementation process (3) might provide data to account for unexplained variance in implementation outcomes.

Data suggests that relationships are critical to implementation success. In a survey of implementation science practitioners, Metz et al. found that almost all survey respondents (99%, *n* = 297) agreed that “relationship quality between the implementation support practitioner and leaders and staff within the service system at the implementing site is critical for the long-term success of change efforts” (11). These results suggest an opportunity for implementation science to consider the relational nature of theories, models, and frameworks and their influence on the process of implementing and sustaining meaningful change (11). Additionally, in a review of the ERIC implementation strategies, Waltz and colleagues identified that the highest proportion of implementation strategies related to the cluster “develop stakeholder interrelationships,” which further reiterates the importance of strategies' utility in relationship development and implementation success (12).

The purpose of this study is to conduct a secondary review of the ERIC implementation strategies (1) and the degree to which they describe relational, operational, or transactional activities to support implementation. Relational strategies are those in which mutual growth, bi-directional or multi-directional exchange occurs, there is shared power, and accountability for growth resides within both parties involved that inherently recognize the vulnerability of the growth-fostering exchange (8). Relational strategies might include co-production, cultivating space for healing, and transforming power dynamics (19). Transactional interactions are unidirectional, influenced by power dynamics, and do not necessitate mutual growth, commitment, or exchange; as a result the power of growth is inherently one-sided. This might include implementation activities such as accreditations, requiring changes, informing partners through one-way communication strategies, or visiting sites. Both types of interactions are relevant in the implementation process, and we recognize that these types are not binary; rather interactions may fall on a continuum between relational and transactional. Two primary research questions guide our review of the ERIC Implementation strategies:

1. How relational, operational, or transactional are the ERIC implementation strategies?
2. From a relational perspective, how are exchanges described within the implementation strategies? What can we learn about how alliances, connections, and power dynamics may be captured in implementation strategies?

## Methods

In order to answer the research questions, a qualitative directed content analysis approach (20) was used to apply Relational Theory to the ERIC implementation strategies (1). Directed content analysis is a useful qualitative approach when an existing theory or prior research exists about a phenomenon that would benefit from further review or description (20). It is guided by a more structured approach (21) in which researchers first identify key concepts or variables as initial coding categories, then develop operational definitions for each category using the established theory (22). We used this methodology because of its utility in applying a high-level application of Relational Theory, which has yet to be used to codify the extensively researched ERIC implementation strategies.

### Development of the relational and transactional strategy continuum measure

Relational Theory suggests that interactions and exchanges are not necessarily binary, and may occur on a continuum. Therefore, in order to code the 73 ERIC implementation strategies from highly relational to highly transactional we developed a categorical coding scheme based on an adaptation of the Relational Intensity Continuum (23) which operationalizes types of relationships in supply chain management from a relational perspective. The Relational Intensity Continuum measure provided a theoretically aligned structure, and we then operationalized each category to distinguish the rating criteria based Relational Theory (8) to focus on aspects of exchanges and interactions including mutuality, connections, and power dynamics. We formulated the categorical coding scheme into the Relational and Transactional Strategy Continuum Measure (Table 1) to detail how relationships, trust, power sharing, and alliances may occur when implementation strategies are used in research and practice. In order to guide reviewers, reflection guiding questions were developed based on the categories' operationalization for the reviewers to consider when rating the strategies. Throughout the coding process, the measure was refined to capture usability feedback from reviewers and consensus meetings.

**Table 1:** Relational and transactional strategy continuum measure (8, 22).

**Highly relational alliance (highly relational)**	**Semi-relational alliance (somewhat relational)**	**Operational alliance (some transactional-some relational)**	**Transactional alliance (somewhat transactional)**	**Highly transactional alliance (highly transactional)**
• Action within the strategy highly reliant on the success of all involved (mutual/bi-directional) • The strategy is highly iterative and can evolve • When implementing the strategy, there are mutual benefits • Accountability for the outcome of the strategy resides in all • Power for implementation of the strategy is shared or power gained by the strategy's implementation is shared • Mutual growth occurs as a result of the strategy's implementation • There is a shared risk/reward of the strategy • Vulnerability may be necessary to implement this strategy well	• The strategy is reliant on the success of those involved, though one party has more responsibility for the success of strategy implementation • The strategy is somewhat interactive in nature • The strategy includes limited exchanges/interactions or are time-limited (~3) • Benefits may be greater for one side of the exchange • Accountability for strategy is shared, though one party involved has more accountability for ensuring strategy implementation • Power is somewhat distributed related to the implementation of the strategy or the outcome of the strategy • When this strategy is implemented, power is mostly accounted for on one side of the relationship	• The strategy involves a working exchange between the parties, with minor technical and relational exchanges • The strategy is slightly interactive in nature • The strategy has minor exchanges (~1–2) when implemented • Benefits to the strategy implementation are technical in nature • The strategy requires limited accountability • Some benefits are experienced among parties, those benefit are more technical in nature	• Some human interaction, though mostly one-sided when implementing the strategy • The strategy is somewhat time-limited (it occurs one or two times when implemented) • Accountability for strategy implementation resides primarily on one side of the relationship (the majority of accountability/responsibility is one-sided) • When this strategy is implemented, power is mostly accounted for on one side of the relationship • Most of the benefits are experienced on one side of the relationship when this strategy is implemented	• The strategy requires limited human interaction and is completely one-sided • The strategy is time-limited (occurs once) • Accountability of the strategy or outcome of strategy resides on one side • Information is “pushed” • Power is one-sided between parties implementing the strategy • One side of the relationship experiences benefits or growth • Risk/reward is limited or one-sided
• Does this strategy require extensive interactions or exchanges among parties? • Does this strategy seem iterative and evolving based on the exchanges? • Does this strategy highly benefit both sides of the exchange? • Does this strategy inherently share power to a high degree? • Does this strategy seem highly relational?	• Does this strategy require several interactions or exchanges among parties? • Does this strategy benefit both sides of the exchange? • Does this strategy have an element of shared power? • Does this strategy seem somewhat relational?	• Does this strategy require some or minor contribution from more than one party? • Does this strategy have elements of both transactional and relational aspects? • Does power fluctuate between parties in this strategy? • Does this strategy seem somewhat transactional and somewhat relational?	• Does this strategy require a few time points to occur successfully? • Does this strategy require minor or some human interaction? • Does this strategy require more “one-sided” implementation with some minor contribution from the recipient? • Does one side benefit more or have more power given the strategy? • Does this strategy seem somewhat transactional?	• Is this strategy time-limited in nature? • Does this strategy require little human connection? • Does this strategy occur uni-directionally? • Is power inherently more one-sided in this strategy? • Does this strategy seems highly transactional?

### Procedures and data analysis

A two-staged deductive and inductive coding process was conducted between three independent reviewers using distinct Excel databases. The excel database included the strategies, definitions, coding categories, and a column to capture reviewer notes and questions. Reviewers had experience in implementation research and practice and knowledge of the ERIC implementation strategies in a variety of human service systems, including child welfare, public health, and early childhood both domestically and globally. First, the reviewers independently coded the same 11 strategies, then combined the individual excel files and met to discuss initial ratings. During the initial coding meeting, there were four strategies that all coders coded the same, and five in which two coders coded the same, which left two strategies in which the three coders coded differently during the first round of coding. Reviewers discussed the codes and coding scheme, then developed a consensus about the codes, and made minor revisions to the continuum categories. Then, to continue to limit individual bias and increase efficiency in rating, the reviewers divided the remaining 62 strategies for review so that each strategy was coded twice. Reviewers independently coded the remaining strategies and met to together discuss any differences in coding through consensus. Thus, even though each of the remaining strategies was coded twice, the entire team of coders discussed each of the ratings for the remaining strategies and developed consensus. During the second consensus meeting, there were a total of 21 differences in coding that the team discussed. The research team also reviewed an Additional File 6 (1) to further consider and categorize the strategies. Finally, an inductive coding process was used to synthesize the ratings, identify commonalities and themes, as well as potential gaps in the strategies compilation. Frequencies can be used in directed content analysis to understand how codes were applied (24) and, in this case, begin to understand how relational, operational, and transactional alliances within implementation strategies can be understood in research and practice.

## Results

**Figure 1:**
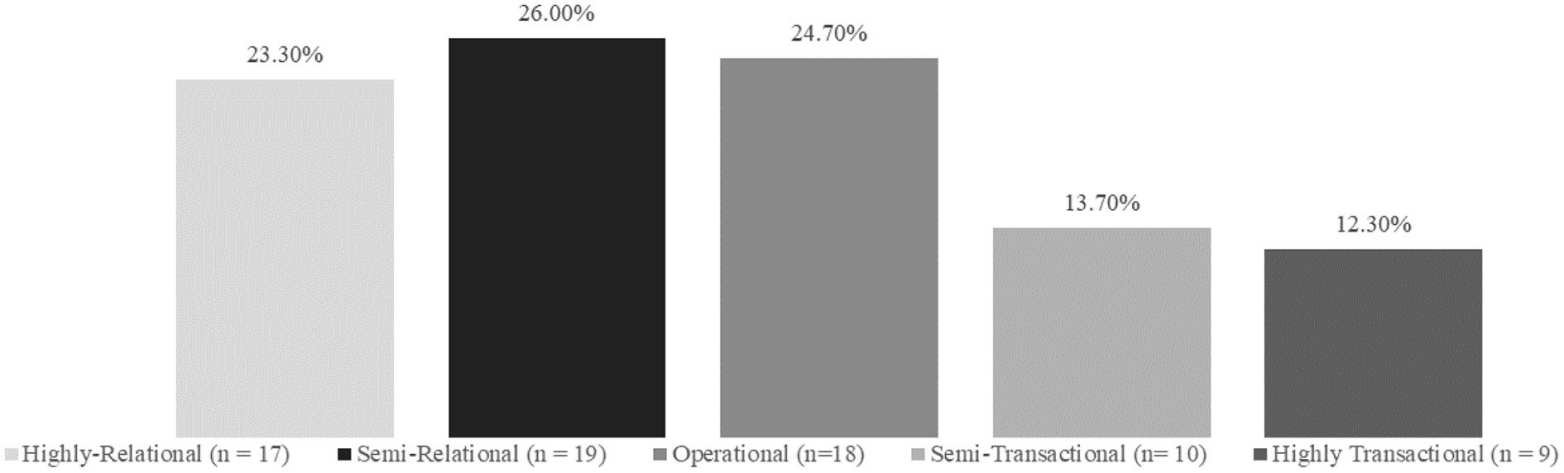
ERIC strategies relational and transactional coding summary results.

**Figure 2:**
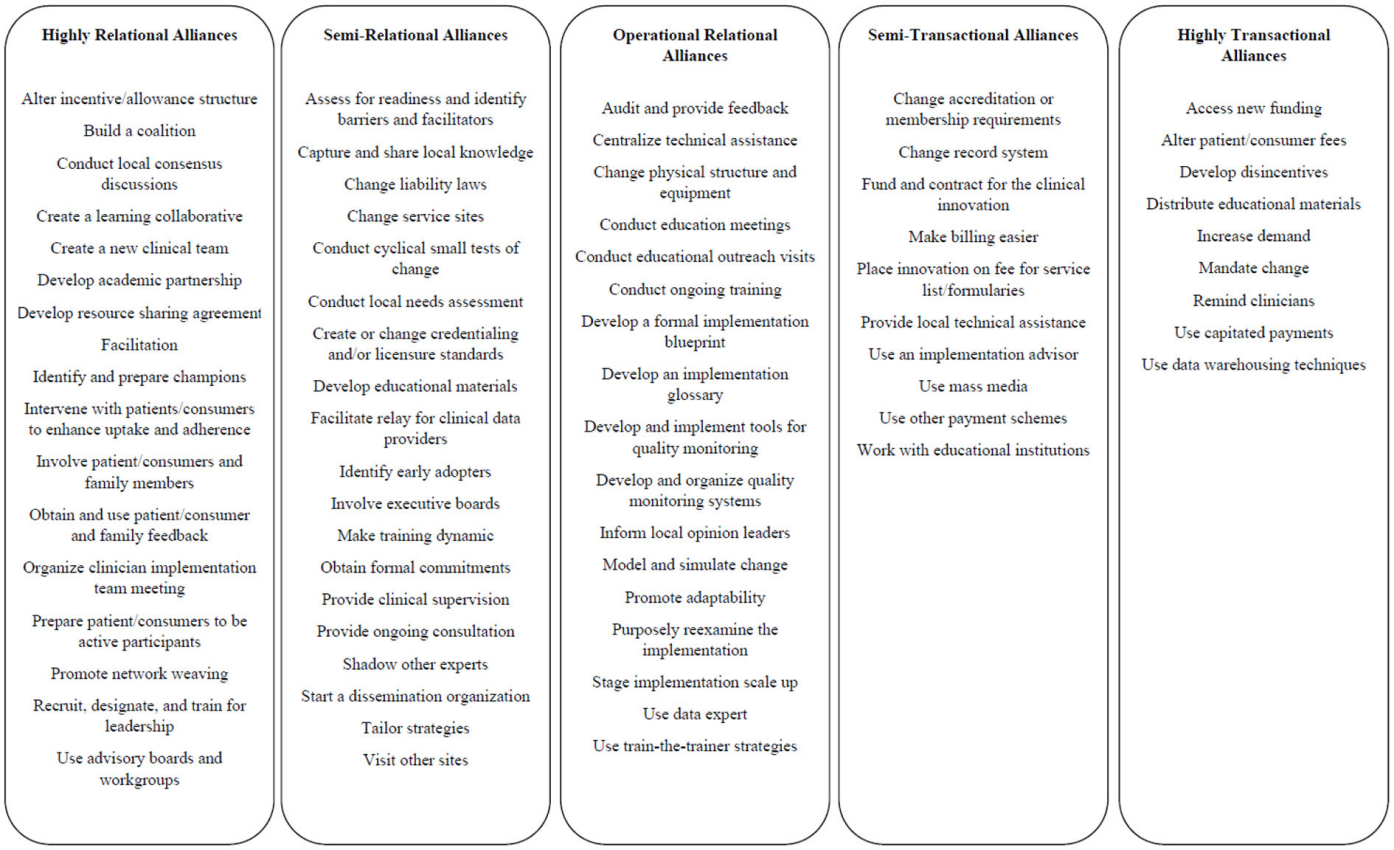
Overview of relational and transactional coding results.

Results from the coding suggested more implementation strategies featured relational alliances (highly relational, *n* = 17, semi-relational, *n* = 19) compared to transactional (highly transactional, *n* = 9, semi-transactional, *n* = 10) and 18 strategies coded as operational alliances, which were strategies that involved a working exchange between parties with minor technical and relational strategies (see Figures 1, 2 for summary of results). Table S1 (in Supplementary files) provides an overview of the ERIC strategies and identified codes. Highly relational alliances included strategies with activities in the definition that suggested multiple or frequent connections between actors, a sharing of power, and the results of the exchange between actors that would suggest robust relationship development. Highly relational alliances had themes related to the creation of groups, such as collaborations, teams, and academic partnerships; strategies in which actors would work together closely, such as conducting consensus discussions, developing resource sharing agreements, facilitating, or promoting network weaving. Partners, partnerships, stakeholder discussions, collaboration, change in roles or involvement of partners, interactive, support, problem-solving, feedback, teams, high-quality working relationships, engagement, and local consensus discussions were all used in highly relational alliances.

Semi-relational alliances included those in which the strategy and definition suggested some degree of mutual or multi-directional exchange occurring between actors that would form a relationship, some potential power sharing, and some degree of iterative development, though to a lesser degree than highly relational. Semi-relational strategies had themes that suggested a change that may require time-limited exchanges and relationship development, such as capturing and sharing local knowledge, changing liability laws, creating and changing credentialing, or making training dynamic. This category also included strategies that would suggest strategies that facilitate improvement of direct practice, such as providing clinical supervision, shadowing other experts, providing ongoing consultation, or visiting other sites. Some of the terms in the strategy or definition of semi-relational included creating, developing, identifying, varying the information, providing, identifying, or starting, suggesting some initial efforts. Highly relational and semi-relational alliances were differentiated by the temporal aspect of the relationship (ongoing or time-limited), the extent to which decision-making authority was fully shared, and the emphasis on mutual accountability for change.

Operational alliances were those in which the strategy suggested a form of working exchange between parties with balanced transactional/technical and relational features. This equilibrium of relational and transactional components is a unique feature of the operational alliance, which may be slightly interactive in nature with minor exchanges and limited accountability. Terms such as “teach them about,” “meet with providers” “plan and conduct,” “develop and distribute,” “inform providers,” “model or simulate,” “monitor progress and adjust,” “shift and revise roles” and “involve or consult experts.

Semi-transactional alliances were those in which there was some interaction among actors, though the definition would suggest it was more one-sided, the alliance was somewhat time-limited, and power within the alliance was mostly accounted for on one side of the relationship. Many of the semi-transactional alliance strategies are related to altering finances in some way – such as billing and payment schemes or marketing, such as use of media or developing dissemination materials for a “push” of information.

Lastly, highly transactional alliances were those in which limited exchange occurred between actors, the strategy was time-limited, and power was one-sided between actors. Highly transactional alliances often related to finances in some way but were described as fully one-sided, such as developing disincentives, altering fees, or capitating payments. A push for information, such as distributing educational materials or requiring shifts in practice or business processes, or “mandating change,” was also associated with highly transactional alliances. These themes suggested that highly transactional alliances are the distinctly one-sided or unidirectional types of exchange and have limited sharing of power.

## Discussion

This is the first attempt to use Relational Theory to offer a critical perspective on implementation strategies. It highlights the potential of ERIC strategies to capture both relational and transactional work of implementation, as half of the strategies (*n* = 36) were coded as highly-relational or semi-relational. This finding emphasizes the importance of relational exchanges in implementation across a broad range of strategies, as the iterative nature of implementation requires adaptive and complex thinking (25). Implementation science has the opportunity to further translate research into routine practice, with particular attention to how relationships, collaboration, and co-design are deployed to improve the implementation process (26). The results suggest an opportunity to consider the relational dynamics within strategy implementation and what types of interactions (e.g., relational, operational, and transactional) may be appropriate given the research and practice goals (10). Additionally, further examination of the relational aspects of implementing particular strategies and their impact on outcomes would enhance our understanding of the value of relational strategies.

Our analysis was limited to the terms and definitions used to identify and explain each of the 73 strategies in the ERIC compilation. As an example of this limitation, for the “Alter incentive/allowance structure” strategy, our team agreed that the definition “work to incentivize the adoption and implementation of the clinical innovation” suggested that it would be relational because some level of exchange would occur in the “altering” and “working to incentivize” aspects of the strategy. After an additional review of Additional File 6 (1) and the ancillary material, this strategy seemed to suggest it would require multi-direction communication and power sharing in the tailoring of incentives or allowance structures. However, it is possible that these activities could be carried out using more transactional methods with less involvement of stakeholders or implementers in the process to alter incentives. Another example of the limitations in our analysis relates to the strategy “Develop academic partnerships” and the definition “partner with a university or academic unit for the purposes of shared training and bringing research skills to an implementation project.” Our study team agreed that the terms “develop, partner, and share training” all aligned with the relational exchange category. However, it is possible that this type of exchange could be carried out in a more transactional method, in which an academic partner provides a “push” of information to a project and reinforces power differentials with local capacity and academic expert “research skills.” Additionally, Additional File 6 (1) acknowledges that “Not all academics have a full understanding of practice-level stakeholder needs and this should be considered while developing partnership.” There is nuance in how implementation strategies are carried out in the real world. Proctor and colleagues provide advanced guidance for strategy reporting that suggests implementation researchers should identify actors, actions, action targets, temporality, dose, implementation outcome, and justification for the strategy's use and implementation (27). From our review, we suggest that adding guidance or language to the strategies themselves describing their relational aspects may improve the utility and uptake of strategies in practice settings as well as how they might be tailored based on the social-relational context. These opportunities are further explored below.

### Enhancing the compilation from a relational lens

Recent research suggests that there are benefits to customizing and tailoring strategies based on a project's goal and the environment from a relational perspective. Some strategies have emerged that are more explicitly relational in nature, as well as enhanced transparency in who is involved in the use of strategies. For example, a study of implementation strategies to improve access to behavioral health services for child welfare-involved youth identified additional strategies with apparent relational aspects, such as obtaining worker feedback and planning for the outcome evaluation (28). As another example, modifications were made to the majority of strategies (52 out of 73), including the deletion of six strategies, and the addition of seven strategies from a study to adapt the ERIC compilation in education. These seven were included in a sister compilation and include relation-focused strategies such as developing a local policy that supports implementation, improving implementers' buy-in, peer-assisted learning strategy pre-correction prior to implementation, pruning competing initiatives, targeting/improving implementer well-being, and test-drive and select strategies (29). Another study in healthcare recommended revisions to four strategy names and 12 definitions, as well as the addition of three new strategies not included in the current ERIC compilation (30). The revisions made in the study often clarified and expanded on who was involved in the strategy process. For example, Perry and colleagues explain that they revised the “organize clinical teams” to “organize implementation teams and team meetings” because “removing the term clinicians allows for a multi-disciplinary team and increases engagement among all team members (p.3).” Additionally, the study also identified three new strategies, including engaging community resources, creating online learning communities, and assessing and redesigning workflow (30). Though the modification of these strategies across the studies can be classified as sub-strategies to existing ERIC strategies, documented benefits from the modification of strategies seem to have potential relational alliance properties. As such, it would benefit implementation research and practice to test and describe the relational aspects of strategies and their impact on outcomes. For example, do particular facilitation or marketing strategies that elevate participant voice and perspective and are highly interactive lead to increased reach or effectiveness of an intervention? Relational Theory in implementation science provides the opportunity to focus specifically on relational exchanges and power sharing associated with implementation strategy deployment.

Findings from a study of implementation support practitioners suggest valuable relational support-system level strategies related to co-creation (co-learning, brokering, addressing power differentials, co-design, tailored support), ongoing improvement (assess need and context, apply and integrate implementation science approaches), and sustain change (grow and sustain relationships, build capacity, and cultivate leadership) (11). Several of these strategies are grounded in relational alliances. Relational alliances are particularly important to the feasibility and transferability of strategies in low-resource communities (31). Here, communication and building on existing networks are critical to promoting equitable implementation and social inclusion. Thus, it may benefit the field to further examine the existing compilation from a relational perspective and consider adding strategies that would support enhanced relationship development and improve implementation strategy deployment through relationship cultivation and nurturing.

### Actors in the strategies

When coding the strategies from a relational perspective, we considered how exchanges and alliances occurred between implementation actors involved in the effective delivery and use of each strategy when considering the second research question in the study. Many of the strategies suggested alliances among actors at the organizational level or in the inner and outer setting of the implementation context (32). Implementation often occurs within the context of relationships at every level, including practitioner-consumer, practitioner-peer-supervisor, and inter-organizationally (33). How do we attend to the need for both transactional and relational strategies among actors at every level of the “system”? Recent studies to assess how the Consolidated Framework for Implementation Research (CFIR) could be applied in implementation efforts in low and middle-income countries suggest the need to expand the framework domains to attend to local application of the CFIR in health care. Findings from this study identified a potential additional domain “Characteristics of Systems” as well as 11 new constructs to increase compatibility in low to middle-resourced countries (34). These findings are relevant to our experience with assessing strategies using a relational perspective and describing how alliances and exchanges occur between different actors within implementation strategies at various levels of the system. Frameworks like the CFIR as well as organizational theories, like Relational Theory could help further identify strategies and expand definitions to highlight how relationships are formed and cultivated through actor exchanges across systems and domains (35).

### Power inherent in implementation strategies

Our review offered an opportunity to consider how power differentials may be present within the compilation through the examination of alliances and exchanges in consideration of the second research question. The Relational and Transactional Strategy Continuum Measure includes reflection on how power differentials may be present within the strategy. Stanton and colleagues provide a useful typology of power generated through the implementation process that can be applied to our review of implementation strategies (36). According to the typology, there are three different types of power: discursive power, epistemic power, and material power. Discursive power describes how dominant perspectives overtake reality and influence actions of others, while epistemic power describes how decisions are made in terms of knowledge, evidence, and perspective, and material power describes the use of resources and control over resources in the implementation process (36). Through a Relational Theory lens, we see preliminary connections to our review of the strategies in terms of how power imbalances may be present in semi-transactional and transactional alliances. Terms included in the compilation and definitions that related concepts such as “experts,” “access,” “fee structures,” “disincentives,” and strategies that were more unidirectional suggested limited power sharing and potential power imbalances in strategy implementation. Issues of power in strategy identification and definition relate to discursive power, which conveys how the language and narratives we use shape our understanding of implementation challenges and processes (36, 37). Discursive power may be present in the inclusion of 73 strategies identified, as the identification, development, and descriptions of strategies were limited to how they were explained and examined in peer-reviewed literature (1). Implementation researchers and practitioners should continue to consider the inclusion of current strategies based on published research and what additional strategies may be relevant in practice as we evolve.

Epistemic power may be present when practitioners and researchers consider how decisions are made regarding the selection and definition of strategies (36, 38). An important consideration when selecting implementation strategies relates to material power, which suggests strategy selection and implementation are impacted by the resources available (36). This is particularly important as we consider implementation in resource-limited settings in which systemic power imbalances may be more pronounced and deeper rooted (31). Though these are initial reflections based on our study process, future research may further consider how Relational Theory can provide an opportunity to consider how exchanges and alliances occur within strategy implementation and the degree to which power is shared or inequitable.

A directed content analysis does have possible limitations that include potential bias when guided by an explicit theory, as researchers might be more likely to find evidence that is supportive rather that non-supportive of a theory (20), and overuse of a theory may potentially blind researchers to a particular phenomenon. To reduce bias, having an audit trail and audit process can be helpful. This was achieved by the development of the rating continuum, and a review and refinement of the continuum ahead of the review, as well as multiple reviewers and consensus development through the coding process (39). A possible shortcoming of the approach is that there was a considerable degree of disagreement among reviewers. Because of the complexity of the task of rating strategies based on limited information, the consensus meetings were an important part of reducing bias in which the three reviewers discussed the ratings, reviewed the additional file 6 and came to a consensus on the ratings of strategies.

The purpose of this study was to examine implementation strategies from a relational perspective. The continuum developed for this study could be used in practice to consider elements of strategy implementation and specify not only the actors, action targets, and outcomes, but also further consideration on how alliances are formed, what strategies may facilitate positive interactions among actors, and the degree to which power dynamics and accountability may be present by using the results of the study to consider potential strategies and how they support relationship development. Understanding the context, nuances, and perspectives of all parties involved in the exchanges is an important aspect of understanding the application of Relational Theory and implementation strategies. Findings from this study could be used early in implementation efforts by practitioners and researchers to consider and select individual or a combination of implementation strategies to strengthen relationships, align with co-design principles, and match the project scope and resources. Table 1 also provides prompts for practitioners and researchers to reflect upon early in project efforts in order to nurture relationships when designing and installing their implementation strategies. This is an important consideration when carefully considering and selecting strategies for implementation and their implications. Implementation researchers can begin to study the differences in effects when strategies are implemented from a transactional, operational, or relational lens and if they impact proximal or distal outcomes differently. Implementation practitioners can further develop and articulate relational strategies that are useful in the real world by using the prompts included in Table 1. Application of Relational Theory using Relational and Transactional Strategy Continuum can also help practitioners and researchers further tailor and implement strategies in ways that support positive alliances in which there are frequent connections, communication, and shared power by considering these important aspects and using the prompts to detail and deploy relational elements in strategy implementation. Additionally, implementation research can continue to elevate the importance of relationships and relational interactions through further application and examination of Relational Theory in studies by focusing on how strategies are deployed with relational properties and if this is related to outcomes or results.

## Data availability statement

The results of the qualitative analysis are provided the Supplementary materials. Any additional material requests can be directed to the corresponding author.

## Author contributions

LB conceptualized the study and wrote the first draft of the manuscript. All authors contributed to the writing and approved the final manuscript.

## Conflict of interest

Author LB was employed by Kaye Implementation and Evaluation. The remaining authors declare that the research was conducted in the absence of any commercial or financial relationships that could be construed as a potential conflict of interest.

## Publisher's note

All claims expressed in this article are solely those of the authors and do not necessarily represent those of their affiliated organizations, or those of the publisher, the editors and the reviewers. Any product that may be evaluated in this article, or claim that may be made by its manufacturer, is not guaranteed or endorsed by the publisher.
